# The Lazarillo’s game: Sharing resources with asymmetric conditions

**DOI:** 10.1371/journal.pone.0180421

**Published:** 2017-07-13

**Authors:** Juan A. Lacomba, Francisco Lagos, Javier Perote

**Affiliations:** 1 Department of Theory and Economic History, University of Granada, Granada, Spain; 2 Department of Economics and Economic History & IME, University of Salamanca, Salamanca, Spain; University of Reading, UNITED KINGDOM

## Abstract

The Lazarillo of Tormes’ picaresque novel introduces a story where two subjects sequentially extract (one, two or three) tokens from a common pool in an asymmetric information framework (the first player cannot observe her partners’ actions). By introducing a reward for both subjects in case that in every period at least one subject had taken one single token, we define an interesting coordination game. We conduct an experiment with 120 undergraduate students to study their behavior in this framework. We find that if the second player is allowed to take more tokens than her partner, then the frequency of cooperators does not seem to be affected by the informational asymmetry. Nevertheless, this asymmetry (i) incentives the second player to use her ‘power of extraction’ while the social externality is still available, (ii) yields to more asymmetric profit distributions when subjects win the social externality and (iii) delays the breach period in case of coordination failure. Furthermore, the first choice of the first player is determinant for getting the reward.

## Introduction

“*Now I want to be generous with you: we’ll share this bunch of grapes, and you can eat as many as I do. We’ll divide it like this: you take one, then I’ll take one. But you have to promise me that you won’t take more than one at a time. I’ll do the same until we finish, and that way there won’t be any cheating.” The agreement was made, and we began. But on his second turn, the traitor changed his mind and began to take two at a time, evidently thinking that I was doing the same. But when I saw that he had broken our agreement, I wasn’t satisfied with going at his rate of speed. Instead, I went even further: I took two at a time, or three at a time—in fact, I ate them as fast as I could. And when there weren’t any grapes left, he just sat there for a while with the stem in his hand, and then he shook his head and said, “Lázaro, you tricked me. I’ll swear to God that you ate these grapes three at a time.” “No, I didn’t”, I said. “But why do you think so?” That wise old blind man answered, “Do you know how I see that you ate them three at a time? Because I was eating them two at a time, and you didn’t say a word*.”

The above paragraph reproduces the original text (translated into English) of *The Lazarillo of Tormes*, *his Fortunes and Misfortunes* (Anonymous, 1554), a major exponent of Spanish picaresque literature of the Golden Age, which presents a clear overview of the society of the sixteenth Century through the eyes of Lázaro of Tormes. Lázaro is a boy who has to earn a living in Salamanca as the apprentice of different masters, the first one being a cunning blind man.

In spite of the huge development in science and technology and the deep changes in the society occurred over the last centuries, this parable maintains all its essence in present days. The story illustrates how the lack of transparency may have an important role in breaking agreements, behaving as a non-cooperative short-term profit maximizer and triggering corruption bubbles (note that Lázaro mimics and overreacts his master immoral actions instead of complaining). There is a long list of examples where agents forget about social externalities and deviate from social norms if they do not feel controlled, e.g., contributors in a typical tax declaration problem, departments charging expenditures on a common budget, firms extracting natural resources, countries deciding about fiscal policies within a monetary union, risk managers deciding about their risk exposure, etc. Nevertheless, in such situations the non-cooperative behavior might not be necessarily a consequence of the lack of transparency or supervision.

To shed light on this issue, we experimentally investigate the effect of asymmetric information between heterogeneous subjects that exploit a common pool (e.g., a natural resource or a budget) through sequential extractions. The topic of voluntary sharing/contribution on common pool resource systems or public goods provision has been recently revisited in experimental literature and explained by indirect reciprocity—e.g. [1–3]. The effects of power asymmetries and punishment in these contexts have been analyzed by [4] and [5], finding that power asymmetries fail to stabilize cooperation when punishment is possible. We propose a different design from those of the traditional prisoner’s dilemma or public goods experiments—see also [6], [7] and [8] for recent studies on cooperation in these contexts. Alternatively, our design investigates the effects of the informational asymmetry on cooperation in a two-player sequential game where the second player has the advantage of playing harder (by extracting more tokens), as well as hiding her actions.

The effects of transparency on conditional cooperation have been studied in other contexts, e.g. public goods [9] or auction markets [10] experiments, though neither of these papers considers asymmetric information within the subjects in the same treatment. Our study is also related to the prominent experimental literature on bargaining games that has analyzed the effect of asymmetric information. For instance, earlier studies [11], [12], [13] or [14] have shown that better-informed agents do not always take advantage of their additional information and thus more information is not always better. [15] also examined the motives that drive individual behavior in bargaining games with asymmetric information. In particular, they analyzed two versions of ultimatum games with one-sided incomplete information. Contrary to the game’s theoretical predictions, they found significant differences in actual behavior between offer and demand games. [16] studied a game where players bargained over chips with different exchange rates and with different information on these exchange rates. They found that when both players are fully informed and first-movers have higher exchange rates, conflicting fairness norms arise, resulting in high rejection rates.

Furthermore, since we deal with heterogeneous subjects (heterogeneity in the sense of different extraction power from the common pool), our work is also related to those studies that investigate the role of asymmetries in sequential settings—see, for instance [17]; [18], [19] or [20] This literature has proved that first movers appropriate more of the common-pool resource (see, for instance, [21]; [22] or [23], [24]). However, in these articles, being first mover is related to a higher capacity of extraction from the common-pool resource. Consequently, the inequality in the rates of resource extraction across participants might be due to either the advantage of moving first or the larger capacity of extracting.

In order to disentangle which of these two asymmetries (information or extraction power) is more relevant, we design an experiment inspired by the aforementioned Lazarillo’s tale where two subjects have to share a given endowment (60 tokens) by means of sequential decisions. In this framework, we introduce incentives for holding a certain sharing rule by giving a final reward (20 extra tokens) to both subjects in case the rule is satisfied in every round of the experiment. The rule requires that at least one of the two subjects has taken a single token, which captures a certain type of “sustainability” in the extraction as in a natural resources problem. Furthermore, and with the purpose of analyzing which of the two aforementioned asymmetries is more relevant, the second player is allowed to take one, two or three tokens in every round whilst the first player is only allowed to take either one or two tokens. The final payoff of every subject is the bunch of tokens taken throughout the experiment plus the final reward in case of success in holding the rule. We consider two treatments: Control treatment, where the first player can also observe the decisions of the second player; and Impunity treatment, where the first player is unable to observe the decisions of her matched partner.

This game involves a subgame perfect Nash equilibrium (SPNE) where in every round the first mover takes two tokens and the second mover responds by taking one single token. However, the capacity of taking three tokens by the second player may represent a credible threat and induce different sharing schemes.

Our results show that most players deviate from the SPNE. In particular, we find that in both treatments second players take advantage of their power of extraction and achieve higher profitability than first players. This occurs not only when coordination fails and they lose the final reward, but also, and more surprisingly, when cooperation holds. This means that the power of extraction seems to be more relevant than the extraction order.

Regarding transparency, we find that non-observability does not seem to distort the level of cooperation (in both treatments around 62% of the groups coordinate their actions so as to be finally rewarded). However, the blindness of the first player has a significant impact on the final distribution of payoffs since second players of cooperators present higher profits in Impunity than Control treatment. Furthermore, Impunity delays the *breach* period (the period in which the condition to win the prize does not hold anymore) compared to the Control treatment.

Finally, we also find that, regardless of the treatment, the *first choice* of the first player is a key determinant for the success in coordination/cooperation. Most of first players that started cooperating (taking one single token in the first period) succeeded in winning the final reward, and, on the contrary, most of first players that started aggressively (taking two tokens in the first round) failed. In fact, both treatments present a positive correlation between the first choice of the first player and the following choices of both players, i.e. the more aggressively first player starts, the more aggressively both subjects behave throughout the experiment, increasing the likelihood of coordination failure.

The rest of the paper is structured as follows: Section 2 describes the experiment and the different strategies and solutions of the game, Section 3 discusses the results of the experiment and, finally, Section 4 gathers the main conclusions.

## Lazarillo’s game and experiment

### Game

Two players have to share *N* tokens in an undetermined number of rounds, *T*, by sequentially extracting them from a common pool (alternatively, the game might be defined by fixing the number of rounds and leaving the number of tokens free). The power of extraction of both players is different. Type A (first mover) can extract either one or two tokens in every round while Type B is allowed to take one, two or three tokens, after Type A has already made her decision, in every period. Therefore if we denote the decision undertaken by subject *i ∈* {*A*, *B*} in period *t ∈* {1, 2, …, *T*} as *d*_*it*_ *∈ D*_*it*_, then *D*_*At*_ = {1, 2} and *D*_*Bt*_ = {1, 2, 3}. Note that, although not explicitly considered in the second player’s options, if in the last round there are no more tokens left for the second player, she is also allowed (forced) to ‘extract zero’.

The game ends when no token remains and the final payoffs of both players depend directly on the number of tokens extracted during the whole game, denoted by $N_{i\;}\;=\;\sum_{t=1}^{T}d_{it}$, *N*_*A*_ + *N*_*B*_ = *N*. In addition, both players may win a reward of *R* tokens in case that at the end of the game the following condition is being satisfied: “In every period *t* at least one of the players had taken one single token”. Note that *R*≥16 is a necessary condition to make both subjects consider coordinating their strategies to win the prize. Therefore the payoff function for player *i* = {*A*, *B*} is:

$$\prod_{i}\;=\;\{\begin{array}{l} N_{i}\;+\;R\;=\;\sum_{t=1}^{T}d_{it}\;+\;R\text{ if }d_{At}\;=\;1\text{ or }d_{Bt\;}\;=\;1\text{for every }t\\ N_{i\;}\;=\;\sum_{t=1}^{T}d_{it\;}\text{ Otherwise} \end{array}$$ (1)

The strategy profiles of every subject may be characterized by a sequence ${\left\{d_{it}\right\}}_{t=1}^{T}$ or a vector $D_{i}^{\prime }\;=\;{\left(d_{i1},d_{i2},\ldots d_{iT}\right)}^{\prime }\in D_{i1}\times D_{i2}\times \ldots \times D_{iT}$. In the next subsection we describe some of the most interesting strategy profiles to be played and their corresponding payoffs in the particular framework of our experiment.

### Experiment

Our experimental design implements exactly the framework described in previous section but setting *N* = 60 and *R* = 20. We applied a between-subjects design to investigate the impact of the informational asymmetry implied by the opacity/transparency of the decisions of first player (Type A) on the decisions of the second player (Type B) and its implications on the coordination/cooperation to win the reward and the final playoffs. For this purpose, we consider two treatments:

**Control treatment (CT)**: Both players can observe the decisions of their matched partners in each round. However, as the game is sequential, Type A only observes the tokens taken by Type B after she has already made her own decision.

**Impunity treatment (IT)**: The first player (Type A) cannot observe the decisions of Type B. We denote it as IT because Type B can take advantage of her power of extraction without being detected by Type A. As a consequence, in this treatment, Type A does not know how many tokens are left in every round. That is, Type A does not know the period in which the game will be over.

In this particular setting we highlight the following strategy profiles as possible outcomes of the experiment:

#### Nash equilibrium strategy profile

The SPNE strategy profile is solved by backward induction. This profile is (2, 1) in every period, i.e. Type A extracts two tokens and thus, Type B takes a single token so as to fulfil the compensation rule the end of the experiment. That is, Type A should always take advantage of being the first mover because he anticipates the fact that in this case Type B would be forced to take one single token. Considering *R* = 20, the final SPNE payoffs would be (60, 40). If Type B deviates from SPNE by extracting three tokens in all rounds, final payoffs would be (24, 36). Thus, if *R* is larger than 16, Type B has no incentive to deviate from SPNE.

**Fig 1:**
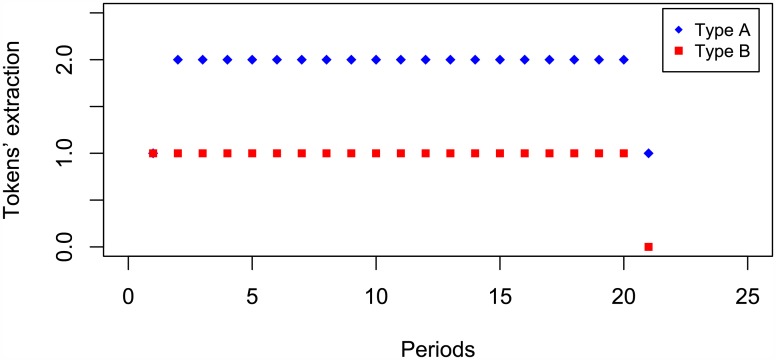
Lab example of Nash equilibrium.

Fig 1 depicts the decisions (X-axis represents periods and Y-axis choices) of a group of the experiment that played SPNE (with the exception of the first observation where both subjects took one token and the last observation in which the first player kept the remaining token). It is noteworthy that this case, as well as the rest of the examples presented in this section, is an outcome of our experiment. In fact, this is the clearest case where subjects played SNPE (the example corresponds to IT).

#### Coordination strategy profiles

We denote by *coordination strategy profile* to every strategy profile such that both agents win the final reward of 20 tokens. Within these profiles payments may vary from (*∏*_*A*_, *∏*_*B*_) = (60, 40) (*∏*_*A*_, *∏*_*B*_) = (35, 65) depending on the times where each subject only takes one token. For instance, Fig 1 illustrates the coordination profile where subjects play Nash equilibrium. In this case, by playing aggressively, Type A forces Type B to play her worst option so as to be rewarded with the 20 extra tokens.

**Fig 2:**
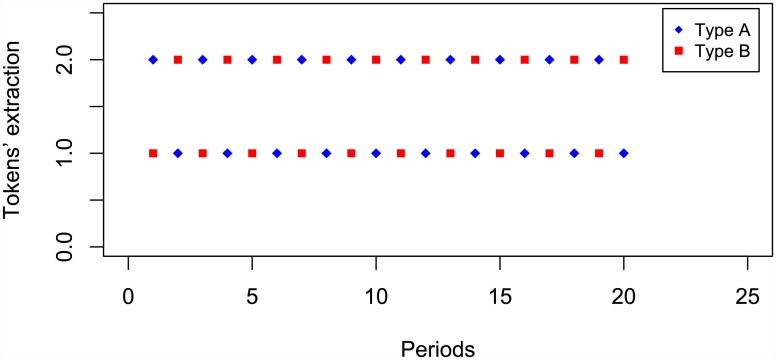
Lab example of egalitarian profile.

Other cases of coordination profiles are those in which both subjects succeed in winning the reward and yield to an egalitarian share ((*∏*_*A*_, *∏*_*B*_) = (50, 50)). Fig 2 displays an example where subjects perfectly coordinate in one of these egalitarian profiles.

**Fig 3:**
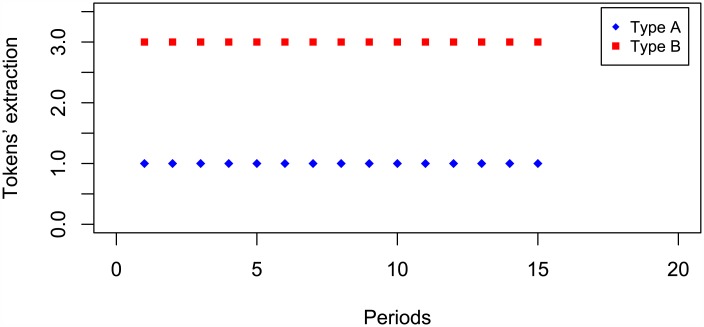
Lab example of coordination profile.

A last example of coordination is displayed in Fig 3. In such figure, the strategy profile represents the opposite situation to the SPNE for both players: Type A (B) takes always one token and Type B takes three tokens during 15 rounds (the example corresponds to IT).

#### Lazarillo’s strategy profile

**Fig 4:**
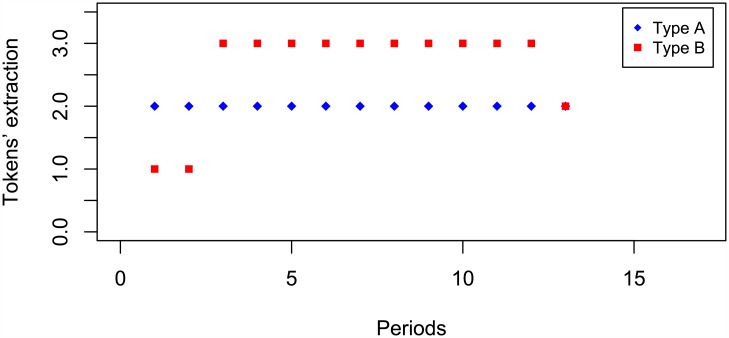
Lab example of Lazarillo strategy profile.

These profiles are those consistent to the story of the Lazarillo of Tormes, i.e., Type A and Type B take, respectively, two and three tokens during 12 rounds. The expected payoff of such a profile is (*∏*_*A*_, *∏*_*B*_) = (24, 36). It is noteworthy that playing this strategy profile might represent a credible threat by player B to avoid that player A exploits her advantage of being the first mover, since Type A has much more to lose than Type B if the Lazarillo’s strategy profile is the final outcome of the game. In fact, the effect of this ‘threat’ on the first mover’s advantage is one of the contributions of this game. Fig 4 illustrates an empirical example of this strategy profile.

### Procedures

The experiment was programmed in z-Tree toolbox [25] and conducted in the Laboratory for Research in Social and Economic Behavior (LINEEX) hosted at the University of Valencia. For each treatment, we organized a session with 60 subjects (30 pairs of independent observations). Hence, a total of 120 undergraduates from various disciplines participated in both experiments. Before the start of the experiment participants were completely informed about the type of experiment and told that they would be rewarded according to their performance. They also provided written informed consent before the experiment had started. The behavioral data, obtained through these participants, were analyzed anonymously and, as the experiments were based on behavioral economics (subjects only make simple decisions about how to share a given amount of tokens), further ethics approval were not necessary.

Participants were randomly assigned in pairs, whose identities were never revealed. To ensure that the data are truly independent across groups, participants were also informed that their matched partner would not change during the experiment. Before the start of a session, participants privately read the instructions (see the Appendix) and their questions were also privately answered. They also had to answer a couple of control questions to make sure that they understood the condition to win the final reward and the experiment did not start until instructions were clear for all subjects. Participants earned experimental currency units (ECUs) that were converted into Euros at a known exchange rate of 0.35€ per token at the end of the experiment. Payment took place privately and the students had to leave the laboratory immediately once they were paid. The average payoff was 14.7€ but it ranged from 7.4€ to 22.8€.

### Hypotheses

The main motivation of the experiment is to discover the impact of asymmetric information on subjects’ cooperation. From the Lazarillo’s tale it is not clear whether Lazaro’s misbehavior was triggered by his master’s blindness or his master’s deceitful behavior. It seems that Lazaro would have tried to cheat his master even if the blind man had behaved honestly and, being aware of it, the blind man aims to indoctrinate his apprentice. As this framework is common to many economic problems, our first hypothesis investigates to what extent impunity hinders cooperation and incentivizes free riding and cheating.

**Hypothesis 1** *The lack of transparency on the second player’s decisions negatively affects cooperation/coordination to achieve a social benefit*.

The focus of the experiment, however, is put on the behavior of the second player. We aim to test how people react to the observation of others’ misbehavior (in terms of deviations from a given rule) and, at the same time, they have impunity for their own actions and are allowed to overreact by extracting more than the others. This makes us formulating the following second hypothesis.

**Hypothesis 2** *The second player exploits her extraction power taking more tokens than the first player and getting higher profitability*.

We also hypothesize that, by reciprocity, transparency makes second players less aggressive (i.e. more cooperative). Consistently, the final distribution of tokens should be more egalitarian in CT than in IT.

**Hypothesis 3** *The lack of transparency leads to more unequal distribution of payoffs*.

On the other hand, CT should accelerate the breach period (period in which the reward is no longer available) when coordination fails, since the aggressive behavior is observed and partner’s tolerance to it reduces.

**Hypothesis 4** *The lack of transparency delays the breach period when coordination fails*.

## Results

The first observation is that the 20 extra tokens reward is won in 18 (19) out of the 30 groups in IT (CT) treatment. Therefore, and regardless of the treatment, around the 62% of the subjects succeed in coordinating their decisions to achieve the social benefit. It is noteworthy that the coordination mechanism rarely follows the SPNE. As a matter of fact, it only happened once (3.3%) in each treatment. Furthermore, the opacity of the second player’s decisions does not seem to significantly hinder cooperation. This finding leads to our first result.

**Result 1** *The one-sided lack of transparency does not reduce the levels of cooperation to get the final reward*.

Although the blindness of Type A does not seem to worsen coordination, the lack of transparency has an impact on the decisions of the players and, consequently, on the final distribution of payoffs. Table 1 displays the main descriptive statistics in both treatments of the experiment (CT in Panel A and IT in Panel B): The average choices of both players, the first choice of the first player, the breach period and the average profit of both players with and without the final reward. All these results are shown for the pooled data and also for the successful (in winning the reward) and unsuccessful groups and while the reward is still available and not. Significant differences in choices and profits of both players within the same treatment (Wilcoxon signed-rank test) are signaled with an asterisk and significant differences in player’s B choices, the profits of both players and the breach period between the two treatments (Mann-Whitney U test) are highlighted with two asterisks.

**Table 1:** Descriptive statistics.

***Panel A*. *Control treatment (CT)***					
	**Pooled data**	**Successful groups**	**Unsuccessful groups**	**Reward available**	**Reward not available**
**Choice A**	1.547*	1.440	1.730	1.510	1.729
**Choice B**	2.041*	1.832	2.412	1.761**	2.543
**First choice**	1.500	1.316	1.818	1.400	1.818
**Breach period**	12.800	--	2.818**	--	--
**Profit A**	26.200*		25.273	26.760	25.273
**Profit B**	33.800*		34.723	33.240	34.723
**Profit+Reward A**	38.867	46.737**	--	41.960	--
**Profit+Reward B**	46.467	53.263**	--	48.440	--
***Panel B*. *Impunity treatment (IT)***					
	**Pooled data**	**Successful groups**	**Unsuccessful groups**	**Reward available**	**Reward not available**
**Choice A**	1.505*	1.430	1.619	1.466	1.668
**Choice B**	2.106*	1.961	2.322	1.929**	2.479
**First choice A**	1.333	1.167	1.583	1.200	1.583
**Breach period**	12.667	--	4.917**	--	--
**Profit A**	25.333*		24.750	25.720	24.750
**Profit B**	34.667*		35.250	34.280	35.250
**Profit+Reward A**	37.333	45.722**	--	40.120	--
**Profit+Reward B**	46.667	54.278**	--	48.560	--

* Denotes that the choices and profits of both subjects are significantly different within the treatment.

** Denotes that the value is significantly different between the treatments.

The results of Table 1 show that the second players extract significantly more tokens than first players in both treatments (Choice B > Choice A, Z = −4.085, and Z = −3.910 for the IT and CT treatments, respectively, all significant with p-value p = 0.000) and, consequently, obtain a higher profitability even when the final reward is not considered (Profit B > Profit A, Z = −3.946, and Z = −3.913 for the IT and CT treatments, respectively, all significant with p = 0.000). This occurs not only, and as expected, when coordination fails and they lose the final reward, but also when cooperation holds and they get it. In other words, the second player exploits her advantage of taking three tokens and get higher profitability. This result does not seem to be affected by the treatment. The lack of transparency of player B, however, does affect her behavior while the reward is still available. In this case, players B decrease significantly their average extraction when their decisions can be observed (from 1.761 to 1.929, Z = −1.741 and p = 0.081), reflecting that transparency makes subjects more prudent, since they do not want to jeopardize the final reward. We summarize these findings in the following result:

**Result 2** *Second players exploit their power of extraction and get a higher profitability than first players*. *However*, *transparency smoothens such behavior while the reward is still available*.

This treatment effect is reinforced by the two additional findings. The first one is that in IT the distribution of profits is less egalitarian for the successful groups in IT (Type A/Type B average profits are 45.722/54.278) than in CT (Type A/Type B average profits are 46.737/53.263). More importantly, 7 out of the 19 succeeding groups (37%) achieved an egalitarian distribution of profits (50 tokens each) in CT whilst only 2 out of the 18 groups (11%) coordinated their strategies to yield the same outcome in IT. The second one is that the average breach period in IT (4.917) is almost two times that of CT (2.818) as a consequence of the opacity of the second players’ actions. The next result summarizes these findings:

**Result 3** *The one-sided lack of transparency yields to a more asymmetric profit distribution for coordination profiles*. *When coordination fails*, *the one-sided lack of transparency delays the breach period*.

Regression analyses and tests in Table 2 support Result 3. Particularly, the last two columns of this table present a logit model for the probability of getting an egalitarian share (i.e. dependent variable is a dummy that takes value one in periods where the reward is available and zero otherwise) and a 2SLS regression on the breach period, where the choices of both subjects were instrumented to control for endogeneity (lagged values of the variables were used as instruments in GMM estimation algorithms, as is usual in dynamic panel models). The models show that variable *treatment* (a dummy variable scoring one in IT and zero in CT) reduces the probability of having an egalitarian share and increases the breach period. Additionally, we also find that both the probability of egalitarian distribution of profits and the breach period depend positively on the variable *patience* (the number of consecutive times in which second player takes one token after observing that first player has taken two tokens). Table 2 also analyses the determinants of keeping the reward available by running GMM regressions, which optimally exploit the moment conditions [26] and present the typical first order autocorrelation in the first differenced residuals. According to these regressions the probability of keeping available the reward depends negatively on the current and lagged choices of both subjects but positively on the second player’s patience (i.e., playing less aggressively favored the success in getting the final reward). These relations are robust through the two treatments.

**Table 2:** Regressions on the determinants of holding available the reward, getting an egalitarian profit distribution and the breach period.

	Available reward (t)		Egalitarian share+	Breach period+
	Control treatment+	Impunity treatment+		
**Choice A (t)**	−.0972* (.035)	−.2868* (.030)		−2.6819 (3.495)
**Choice A (t − 1)**	−.1865* (.034)	−.4140* (.070)		
**Choice B (t)**	−.0338* (.011)	−.0611* (.011)		1.902 (2.226)
**Choice B (t − 1)**	−.0855* (.017)	−.1535* (.033)		
**Patience**	.0762* (.006)	.0543* (.090)	1.027* (.313)	1.832* (.331)
**First choice A**			4.667 (3.936)	
**Treatment**			−8.467* (2.094)	.6268* (.346)
**Intercept**	.8407* (.165)	1.8691* (.242)	−14.191* (5.645)	
**Arellano-Bond AR(1)**	−2.77*	−2.30*		
**Sargan (Overidentified restrictions)**	1268.31*	1269.07*		
**Number of obs**.	432	428	569	322

^+^Robust standard errors in parentheses.

* denotes significance at 5% confidence.

Finally, we investigated on the determinants of the players’ choices. Table 3 displays the GMM regressions on the choices of both players in both treatments. The most relevant result is that the first choice of the first player positively affects the choices of both players during the whole game with the exception of the choices of Type A in IT (since in this case Type A does not observe her partner’s responses). This means that the first choice of the first player determines game dynamics. In fact, 27 out of the 34 (79, 4%) of first players that started cooperating (taking one token in the first period) succeeded in winning the prize. This effect is even stronger in CT (12 out of 14, 85, 7%) than in IT (15 out of 20, 75%). On the contrary, 16 out of 26 (61, 5%) of the of first players that started aggressively (taking two tokens in the first round) failed. This evidence is stronger in IT (7 out of 10, 70%) than in CT (9 out of 16, 56, 2%).

**Table 3:** Regressions on the determinants of choices.

	Control treatment (CT)		Impunity treatment (IT)	
	Choice A (t)	Choice B (t)	Choice A (t)	Choice B (t)
**First choice A**	.1381** (.040)	.2688** (.062)	.0635 (.049)	.2855** (.064)
**Choice B (t − 1)**	.1671** (.021)		.0655** (.024)	
**Guess error A (t − 1)** ^+^	.0604* (.031)		.0092 (.027)	
**Choice A (t)**		−.8641** (.069)		−1.1782** (.068)
**Guess error B (t − 1)** ^++^		−.1777** (.072)		−.0976* (.062)
**Intercept**	.9708** (.075)	2.9334** (.133)	1.2874** (.084)	3.4929** (.131)
**Arellano-Bond AR(1)**	−3.39**	−4.10**	−3.38**	−3.14**
**Arellano-Bond AR(2)**	1.09	1.63	1.59	3.10**
**Sargan (Overidentified restrictions)**	435.09**	380.19**	710.09**	588.86**
**Number of Obs**.	484	484	475	475

^+^ Defined as the absolute value of the difference between Type A’s guess about Type B’s choice and Type B’s true choice.

^++^ Defined as the absolute value of the difference between Type A’s guess about Type B’s choice and Type B’s true choice in the next period.

Robust standard errors in parenthesis.

* and ** denote significance at 10% and 5% confidence, respectively.

**Result 4** *The first choice of the first player is positively correlated with the choices of both players during the game and represents a signal for the willingness to cooperate*.

Furthermore, Type A’s choices in both treatments depend positively on Type B’s actions in the previous period (conditional cooperation) and on the errors in guessing Type B’s choices (i.e. deception on Type B’s expected choices makes Type A play harder). The latter assessment is based on the guesses about partner’s choices that were elicited in every period during the experiment (before taking their decisions subjects were asked to guess the other player’s action). These guesses were not incentivized. Observe that the positive correlation between the choices of Type A and the lagged choices of Type B in IT implies that Type B is capable of predicting the actions of Type A to some extent (as in the Lazarillo’s story). The choices of Type B, however, depend negatively on the contemporaneous choices of Type A in both treatments since Type B can always observe Type A’s movement and her decision is conditioned on it. The negative correlation is likely induced by her attempts to keep the reward available. Finally, misperceptions about the expected choices of Type A affect negatively to her choices (although this effect is weaker in IT).

## Conclusions

There exist many different situations where if agents coordinate their actions according to a certain rule of sustainability they get a social externality (e.g., firms extracting natural resources, countries deciding about fiscal policies within a monetary union, departments charging expenditures on a common budget or risk managers deciding about their risk exposition). In all of these situations it seems that the asymmetric lack of transparency makes the agents more aggressive on their decisions (short term profit maximizers) and put the social benefits of coordination/cooperation under jeopardy. We designed a new experiment to study the effects of the asymmetric information on the actions of individuals facing such problems. Unlike other related games based on the prisoner’s dilemma, the induced game is sequential, although we endowed the second player with the power to play harder than her opponent (by taking more tokens) to offset to some extent her disadvantage of being the second mover. This is a key issue of our experiment since we focus on the reactions of the second player on the first player’s actions when her own actions can be hidden.

The experiment, inspired by the tale of the Lazarillo of Tormes, asks two agents to sequentially extract tokens from a finite common pool until there is no token left. Since we are interested in analyzing which asymmetry is more relevant, either the advantage of moving first or the larger capacity of extracting, we let the second player extract more tokens than the first player in every round (one, two or three, while the first player is only allowed to extract one or two). In the Control treatment there is full information about the actions but in the Impunity treatment only the second player can observe the decisions of her partner. We also include a valuable reward for both subjects (social externality) in case subjects coordinate their actions in such a way that in every round at least one of them had taken a single token.

Under these conditions we observe that the larger capacity of extracting outweighs the advantage of being the first mover and thus second players achieve higher profitability. This occurs regardless of the failure or success in coordinating to get the final reward, as well as in both treatments. Although, the one-sided lack of transparency does not seem to significantly affect the level of cooperation, it plays an important role in three different dimensions: (i) It increases the ‘abuses’ of the second player while the reward is still available, (ii) it yields more asymmetric profit distributions when subjects succeed in coordination (iii) it delays the *breach* period in case of coordination failure. Furthermore, we find that the *first choice* of the first player determines the behavioral dynamics of the game. Most of first players that started cooperating (aggressively) succeed (fail) in winning the prize.

In summary, the Lazarillo’s game results to be a useful scheme to understand cooperation in contexts of asymmetric informational conditions and highlights the importance of transparency in decision-making. The experimental evidence reflects that transparency reduces the abuses of the more informed subjects and induces more egalitarian profit distributions.

## Appendix

### Instructions (Impunity Treatment)

Thank you for participating in this experiment. The purpose of this experiment is to study how people make decisions in a particular context. The instructions are simple and if you follow them carefully you will be able to win money in cash and confidentially (nobody will know the payments of the other participants). In the experiment there are neither correct nor incorrect answers. Do not think that we expect a particular behavior. However, notice that your decisions will affect your payments. If you raise your hand someone will answer your questions, but no other communication is allowed during the experiment.

1. In this experiment there is a participant A and a participant B. Each participant A will be randomly matched with the same participant B during the whole experiment.
2. At the beginning of the experiment you will know whether you are a participant A or B, which will be randomly assigned by the computer. The identity of your matched partner will be anonymous during and after the experiment.
3. The experiment has a number of rounds in which both partners will have to share 60 tokens.
4. Every round has two steps.
5. Step 1: Participant A may take either one or two tokens in every round.
6. Step 2: Participant B, after observing the action of participant A, may take either one, two or three tokens in every round.
7. If in every round at least one of the participants had taken one token, then both participants will get 20 extra tokens at the end of the experiment.
8. Participant A will never know the number of tokens taken by participant B in each round.
9. At the end of the experiment you will be privately paid your earnings. Your final payment will be the sum of the tokens you had accumulated during the experiment. The tokens will be converted to Euros at the rate 1 token = €0.35.

#### Summary

- If you are the Participant A… you can take one or two tokens per round.
- If you are the Participant B… you can choose one, two or three tokens per round and you can observe the tokens taken by Participant A.
- In every round if at least one of the participants have taken only one token then both participants will receive 20 extra tokens at the end of the experiment.

#### Questionnaire

To make sure that you have understood these instructions and before starting the experiment you have to answer a simple questionnaire and then you will be able to proceed with the experiment in your computer.

1. If Participant A has taken a total of 22 tokens and Participant B has taken a total of 38 tokens and, at least in one round none of the participants had taken just one token, then which is the final payoff (in tokens) of both participants?
  *Participant A* = ____ tokens
  *Participant B* = ____ tokens
2. If Participant A has taken a total of 22 tokens and Participant B has taken a total of 38 tokens and, in all rounds at least one of the participants had taken only one token, then which is the final payoff (in tokens) of both participants?
  *Participant A* = ____ tokens
  *Participant B* = ____ tokens

## Supporting information

S1 File Experimental data. Data obtained from the experiment.
(ZIP)
